# Finding a Balance: How Diverse Dosage Compensation Strategies Modify Histone H4 to Regulate Transcription

**DOI:** 10.1155/2012/795069

**Published:** 2011-10-19

**Authors:** Michael B. Wells, Györgyi Csankovszki, Laura M. Custer

**Affiliations:** Department of Molecular, Cellular, and Developmental Biology, University of Michigan, Ann Arbor, Michigan, MI 48109-1048, USA

## Abstract

Dosage compensation balances gene expression levels between the sex chromosomes and autosomes and sex-chromosome-linked gene expression levels between the sexes. Different dosage compensation strategies evolved in different lineages, but all involve changes in chromatin. This paper discusses our current understanding of how modifications of the histone H4 tail, particularly changes in levels of H4 lysine 16 acetylation and H4 lysine 20 methylation, can be used in different contexts to either modulate gene expression levels twofold or to completely inhibit transcription.

## 1. Need for Dosage Compensation

Proper chromosome dosage is essential for the viability and fitness of an organism [1]. Most variations in chromosome quantity (aneuploidies) are inviable [1]. Some aneuploidies are tolerated, but result in severe developmental phenotypes, including Down syndrome, trisomy 21 [1]. However, a difference in sex chromosome copy number must be accommodated across many species. Sex can be determined by sex chromosomes, where one sex is homogametic for the sex chromosome, while the other is heterogametic. In the XY sex chromosome system, females have two X chromosomes, and males are XY or XO. In the ZW system, males are ZZ, and females are ZW. As a consequence of these differences, the heterogametic sex is functionally monosomic for the sex chromosome. The X and Z chromosomes encode genes involved in many processes required for life, not just sex-specific processes. To cope with this disparity, dosage compensation balances the expression of the sex chromosomes to the diploid autosomes and equalizes sex chromosome expression between males and females. 

**Figure 1:**
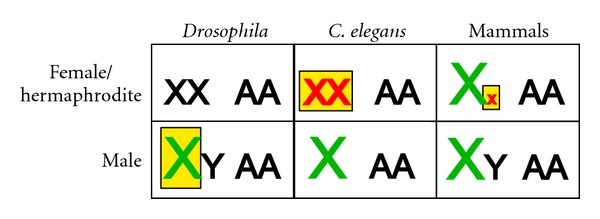
X chromosome dosage compensation. Dosage compensation balances expression of the X chromosomes between males and females and equalizes expression between the X and autosomes. In male flies, the single X chromosome is upregulated. *C. elegans *upregulates the X chromosomes in hermaphrodites and males, and the dosage compensation complex functions in hermaphrodites to downregulate transcription two-fold. The X chromosomes are upregulated in female and male mammals, but one X chromosome is inactivated in females. Green text indicates upregulation, and red text indicates downregulation. Yellow boxes depict chromosomes that are targeted by specific dosage compensation mechanisms.

Dosage compensation has been studied in mammals, worms, flies, and birds. These organisms all cope with sex chromosome imbalance between males and females; however the mechanisms and machineries that they use differ widely (Figure 1). In the fly *Drosophila melanogaster*, XY males upregulate their single X chromosome twofold [2]. This process accomplishes both goals: it balances expression of the single X with autosomes and also equalizes X-linked gene dosage in the sexes. Although less well understood mechanistically, X chromosome upregulation is thought to occur in both sexes in mammals [3, 4]. While this balances the genome in XY males, it causes overexpression of the X chromosomes in XX females. A second (and better understood) mechanism then inactivates one of the two X chromosomes in females, thereby equalizing X expression [5]. In the nematode *C. elegans,* the X chromosomes are thought to be upregulated in both XO males and XX hermaphrodites [3] then downregulated two-fold in hermaphrodites only [6]. In birds, dosage compensation occurs regionally on the Z chromosome. This partial dosage compensation increases expression of required genes in ZW females [7]. 

The dosage compensation strategies outlined above include two-fold upregulation, two-fold downregulation, and complete transcriptional silencing. Interestingly, one feature of chromatin appears to be involved in all of these mechanisms: a difference in the level of histone H4 lysine 16 acetylation (H4K16ac) on the dosage compensated sex chromosome(s). In this paper, we will describe our current knowledge of H4K16ac and its role in modulating the structure of chromatin and regulating transcription. We will then describe how changes in levels of this modification correlate with transcriptional regulation in a diverse array of dosage compensation strategies.

## 2. Nucleosome Structure and Histone Modifications

Chromatin is a dynamic and flexible structure that not only serves to package DNA into higher-order structures, but also regulates access to the DNA. In the nucleosome, 147-bp of DNA wraps around an octamer of histone proteins, composed of two each of histones H2A, H2B, H3, and H4 [8]. Histones H2A and H3 may be replaced by a histone variant protein [9]. The N-terminal tails of the histones extend from the nucleosome core and can be posttranslationally modified by phosphorylation, methylation, ubiquitination, and acetylation [10, 11]. Modification of the histone tails influences the interactions of neighboring nucleosomes and access of regulatory proteins.

Nucleosome structure affects higher-order folding of the chromatin fiber. High-resolution structure analysis of the nucleosome has provided insights into the interactions between neighboring nucleosomes. Histone H4 tails are highly basic and are thought to bind to an acidic patch in the H2A-H2B dimer in the neighboring nucleosome [12]. Binding across nucleosomes suggests that the histone H4 tail is more important for interactions between nucleosomes than for interactions with other histones within the same nucleosome. Computational modeling has demonstrated that the histone tail forms an *α*-helix centered around lysine 16 [13]. In its unmodified form, the histone tail *α*-helix aligns basic charges in one direction, which allows a perfect fit and strong interaction with the acidic patch in the neighboring nucleosome [13].

## 3. H4K16 Acetylation

Histone H4 can be acetylated on lysines 5, 8, 12, and 16. Studies using site-specific antibodies have indicated that H4K16ac is usually present in the monoacetylated form of the H4 tail [14–16]. The order of acetylation of the other lysines in preexisting H4 tails proceeds in the N-terminal direction, such that K12 is acetylated second, then K8, and finally K5 [17]. In newly synthesized histone tails, K5 and K12 are acetylated first [18]. The pattern of acetylation of the H4 tail is the same in human, mouse, yeast, and *Tetrahymena*, demonstrating the universality of the H4 acetylation mechanism [19]. 

Regulation of K16 acetylation is unique from the other lysines of histone H4 [20], highlighting the importance of this particular modification. Regulation of H4K16ac is achieved by the balance between MYST domain histone acetyltransferase (HAT) and class III histone deacetylase (HDAc) (Sir2 family) activities [21]. However, recent evidence suggests that this balance is quite complex. Lu and others have shown in HeLa cells that SIRT1 (a Sir2 homolog) activity is needed to limit hMOF (MYST HAT) autoacetylation to allow hMOF to bind DNA [22]. Further, this work suggested that direct regulation of MYST HAT activity is conserved across many species, including additional mammalian systems, *C. elegans*, and *D. melanogaster* [22]. This mechanism suggests that both direct and indirect means are used by the deacetylase SIRT1 to regulate histone acetylation.

**Figure 2:**
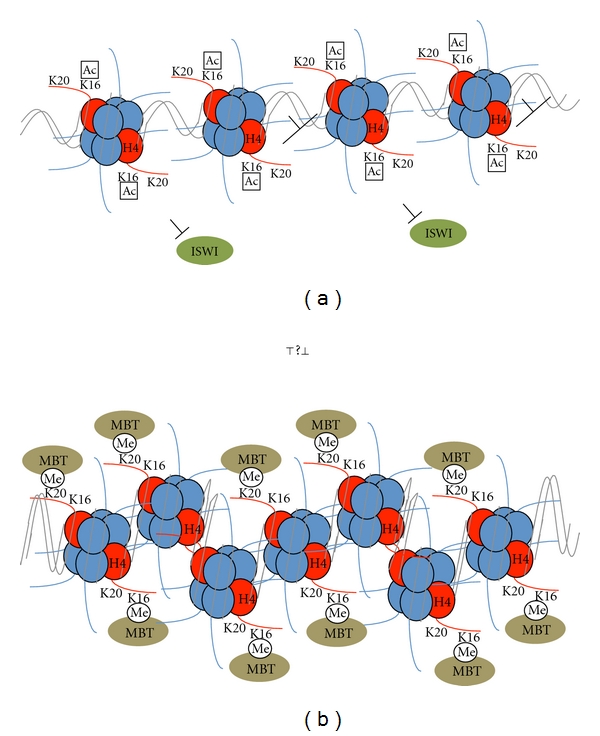
A model illustrating the antagonistic effects of H4K16ac and H4K20me1 on chromatin packaging. (a) Chromatin acetylated at H4K16 is loosely packed, due partially to charge neutralization, and partially to effects on interactions with chromatin modifying proteins, such as inhibition of chromatin remodeling by ISWI. (b) Chromatin methylated at H4K20 is tightly packed. In some systems, H4K20me1 and H4K16ac antagonize each other (see text). H4K20me1 also binds to MBT domain containing proteins, which may facilitate chromatin compaction.

H4K16ac is thought to play a central and unique role in modulating chromatin structure (Figure 2(a)). It is unique among posttranslational histone modifications in that it directly affects the structure of the chromatin fiber. Acetylation of K16 decreases the positive charge of the histone tail, destabilizes the *α*-helical conformation of the tail, and disrupts the interaction of the tail with the acidic patch on the H2A/H2B dimer surface [12, 13]. Therefore, K16 acetylation triggers the unfolding of chromatin by disrupting the interactions between neighboring nucleosomes. Sedimentation assays that evaluate the degree of nucleosome array folding or intraassociation, which mimics formation of the 30-nm fiber, have demonstrated that H4K16ac inhibits nucleosome array folding [23, 24]. Tetra-acetylated H4 dramatically inhibits intraarray folding, more than H4K16ac alone, suggesting that additional acetylation of the H4 tail beyond H4K16 creates an environment even more disruptive to nucleosome folding [23, 24]. Acetylation of K16 also perturbs the divalent cation-induced self-aggregation of nucleosome arrays, thought to mimic higher order folding, or inter-array interactions [23, 24]. Mutation of K16 to a glutamine mimics acetylated lysine but does not cause decompaction of a nucleosome array, indicating that K16 is critical for decompaction [25]. Higher acetylated forms of the H4 tail further prevent self-aggregation of arrays [23].

H4K16ac not only affects nucleosome interactions, but also affects interactions of the nucleosome with chromatin-associated proteins. ISWI is a member of the family of chromatin remodeling ATPases that promotes regularity of nucleosomes and chromatin folding. ISWI binds to amino acids 17–19 within the H4 tail, and this binding stimulates ISWI activity [26–28]. Acetylation of the nearby lysines 12 and 16 impairs the ability of ISWI to recognize its target binding site to compact chromatin and to slide nucleosomes along DNA [24, 27, 28]. 

## 4. H4K20 Methylation Antagonizes H4K16 Acetylation

The fifth lysine residue on the H4 tail, K20, can be mono-, di- or trimethylated. Histone H4 lysine 20 monomethylation (H4K20me1) is established by the histone methyltransferase PR-Set7/Set-8 [29, 30], and Ash1 also monomethylates H4K20 in *Drosophila* [31]. Di- and trimethylation of H4K20 (H4K20me2/3) is accomplished by SUV4-20 [32, 33]. H4K20 methylation antagonizes H4K16ac and is therefore important for controlling gene expression [30, 34, 35]. In *in vitro* assays, H4K20 monomethylation antagonizes acetylation of H4K16 and vice versa [30], and levels of these two marks inversely correlate during cell cycle progression in human cells [35]. However, other studies showed substantial overlap between H4K20me1 and H4K16ac at the *β*-globin locus, indicating that these marks are compatible in some circumstances [36]. The action of H4K20me1 on chromatin is also context dependent. H4K20me1 correlates with active transcription in some contexts [37–40], while in others it is associated with repressed genes [41–44]. For the purposes of this paper, we will focus on H4K20me1's repressive action because of its role in antagonizing H4K16ac.

H4K20me1 can induce chromatin compaction (Figure 2(b)). The mark is found in the same compartment as other repressive marks in many systems and is proposed to regulate the packaging of chromatin into facultative heterochromatin and serve as an intermediary toward H4K20me3 enrichment in constitutive heterochromatin [11, 32, 43–48]. Consistent with a role in chromatin compaction, depletion of PR-Set7 results in decondensed chromosomes [49]. Binding of MBT (malignant brain tumor) domain-containing proteins to the H4K20me1 mark contributes to chromatin compaction [50, 51]. The mechanism of chromatin compaction by MBT domain-containing proteins is not completely understood, but it may involve binding to multiple nucleosomes and DNA bending or bridging of neighboring nucleosomes by dimerization of the MBT domain [51–53].

## 5. The Effect of H4K16ac/H4K20me1 on the RNA Polymerase II Transcription Machinery

**Figure 3:**
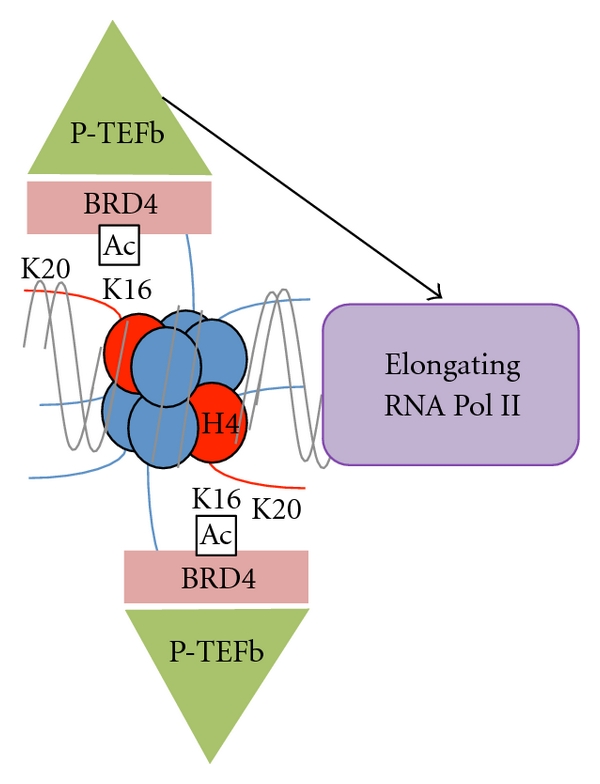
A model of transcriptional regulation by H4K16ac. H4K16ac recruits the transcription elongation factor P-TEFb through the transcriptional coactivator BRD4. P-TEFb phosphorylates RNA Pol II, signaling the transition to productive elongation.

In addition to affecting chromatin structure, H4K16ac and H4K20me also regulate the RNA Polymerase II machinery directly. Transcription initiation is a highly regulated process [54]. After initiation of transcription, RNA Polymerase II stalls just downstream of the transcription start site in many highly regulated genes [55]. Stalled polymerase remains at this site until elongation factors, such as P-TEFb, are recruited to facilitate transition to productive elongation [55–57]. P-TEFb recruitment to active loci is an intricate process, involving release of P-TEFb from a sequestration complex by activators including BRD proteins, which are recruited to RNA Pol II and chromatin by H4K16ac [58, 59]. Recruitment of BRD4/P-TEFb to the chromatin occurs by recognizing the combination of H4K16ac and H3S10 phosphorylation, which provide a binding platform for the complex, at least at the FOSL1 gene (this model is shown on Figure 3) [60]. 

The role of H4K16ac in gene expression has been studied extensively in budding yeast [61, 62]. While H4K16ac is present throughout most of the genome, H4K16 is hypoacetylated at silenced loci, including the mating type loci and telomeric regions [63]. The Sir2, 3, and 4 proteins form a complex essential for transcriptional repression at silenced regions [64]. The Sir complex mediates deacetylation of H4K16 in neighboring nucleosomes through Sir2 action [65, 66]. Deacetylation of H4K16 by Sir2 represses transcription by reducing RNA Pol II promoter occupancy [67] or blocking access of capping enzymes and elongation factors to RNA Pol II, reducing transcriptional elongation [68, 69].

Acetylation of H4K16 is important for transcriptional activation, while H4K20 methylation is suggested to have direct repressive effects on transcription in certain contexts. Trimethylation of H4K20 has been proposed to limit RNA Pol II transcription by blocking H4K16ac and P-TEFb recruitment [70]. PR-SET7 and L3MBTL1 interact directly to repress transcription of a reporter gene, suggesting that H4K20 monomethylation is directly required for transcription repression [71]. Loss of H4K20 monomethylation in multiple studies has indicated the role of this mark in silencing. Deletion of PR-Set7, the H4K20me1 HMT, in flies causes reactivation of genes located in heterochromatin and which would normally be silenced [42]. Furthermore, knockdown of PR-Set7 results in decreased H4K20me1 and an approximately two-fold increase in expression of H4K20me1-associated genes in mammalian cells [41]. H4K20 methylation and H4K16ac have opposing effects on regulation of transcription and transcription machinery, as expected given their mutual antagonism.

## 6. Involvement of H4K16 Acetylation in Dosage Compensation Mechanisms

### 6.1. Upregulation of Gene Expression: Flies and Birds

Fly dosage compensation is accomplished by two-fold upregulation of the single male X chromosome by the *male-specific lethal* (MSL) complex, composed of the proteins MSL1, MSL2, MSL3, MLE, and MOF, and two noncoding RNAs, roX1 and roX2 [2, 72]. The MSL complex specifically binds the X chromosome. The current model of MSL binding to the male X chromosome includes a two-stage process: first, MSL-1 and -2 bind and load at ~150 high affinity (chromatin entry) sites; then, the other proteins localize and facilitate spreading of the complex to many more sites of action across the single male X chromosome [73, 74]. MSL complex loading involves a DNA sequence motif, GAGAGAGA [73]. Models for the spreading of the MSL complex include recognition of cotranscriptionally deposited H3K36 methylation [75, 76], MOF-dependent acetylation/deacetylation cycles tuning MSL-3 activity [77], and binding of specific chromatin features by the MRG domain of MSL-3 [78–80]. The histone acetyltransferase subunit of the MSL complex, MOF, acetylates histone H4K16 leading to an enrichment of this mark on the X [81–84]. By contrast, levels of H4K20me1 are low on the male X [30], although some level of H4K20me1 appears to be necessary for spreading of the MSL complex [79, 80]. JIL-1 kinase, which phosphorylates H3S10 and synergizes with H4K16ac action, also contributes to fly dosage compensation [85–87].

There is also evidence that ISWI, whose binding to chromatin is blocked by H4K16ac, may play a role in fly dosage compensation. X chromosome bloating, which indicates severe decondensation, was seen upon perturbation of the ISWI-containing NURF complex [88, 89]. Blocking H4K16ac in males suppresses X chromosome defects seen in ISWI mutant male flies [28]. Conversely, aberrant overacetylation of H4K16 in ISWI mutant females caused chromosome decompaction defects identical to those seen in ISWI mutant males, especially on the X chromosomes, and broad-reaching gene misexpression [28, 90]. Increased MOF expression also strongly enhances the ISWI loss phenotypes [28].

How does the MSL complex enhance transcriptional output? MSL localization and MOF-dependent H4K16ac are biased toward the 3′ end of gene bodies, which suggests that fly dosage compensation might regulate transcription elongation [75, 91]. Recent work utilizing global run-on sequencing analysis has yielded compelling evidence that dosage compensation in flies is achieved by increased transcription elongation of male X chromosome genes [91]. Other studies have provided further hints that males dosage compensate by increasing transcriptional elongation. The viability of males was greatly affected by knockdown of the elongation factor dELL in flies [92]. The MSL complex chromatin entry site binding motif is a GA-rich sequence [72, 73]. GAGA factor binds to a GAGA motif and helps to release paused polymerase at many genes [93]. Mutations in the GAGA factor gene disrupt dosage compensation in *Drosophila* [94]. JIL-1, the kinase known to play a role in fly dosage compensation, is also involved in transcriptional pause release [60]. The conclusion that fly dosage compensation acts at the level of transcription elongation is consistent with the role of H4K16ac in facilitating release of paused polymerase in *Drosophila* and the other systems described previously.

Like flies, birds regulate expression from the sex chromosome by upregulation. In birds, males (ZZ) are the homogametic sex, and females (ZW) are the heterogametic sex. However, despite the Z chromosomal imbalance between avian males and females, there is no evidence that birds have a chromosome-wide dosage compensation mechanism [95–97]. Rather, it appears that birds use region- or gene-specific methods to balance Z gene expression. 

When comparing the expression ratio of genes along the Z chromosome between ZZ male and ZW female chickens, one area displays clear female bias [98]. This region is the MHM (male hypermethylated) locus and is enriched in compensated genes. A non-coding *MHM* RNA is expressed specifically in females [99]. Because the region is hypermethylated in males, it is not transcribed. H4K16ac is strikingly enriched in one area of the nucleus in a female-specific manner [100]. Increased acetylation of H4 at K5, K8, and K12 was also noted in females, although to a lesser extent than acetylation of H4K16. Further analyses demonstrated that the area of increased H4K16ac corresponds to the MHM locus [100]. The enrichment of H4K16ac at the dosage-compensated region in ZW female chickens resembles the enrichment of H4K16ac on the X chromosome in XY male flies, although only at one locus and not chromosome-wide. However, the mechanism of partial dosage compensation may be similar to chromosome-wide compensation, and regional acetylation of H4K16 may allow for increased expression of Z genes sex specifically. 

### 6.2. Transcriptional Downregulation: Worms

Dosage compensation in the worm uses a mechanism different from flies and birds. Upregulation of the X is thought to be non-sex-specific, creating a need to dampen X-linked gene expression in the hermaphrodite. This is achieved by twofold downregulation of each hermaphrodite X chromosome, equalizing expression with that of the single male X [6, 101–107]. This is achieved by the dosage compensation complex (DCC), which is composed of two parts. The first part is condensin I^DC^, which shares four of five subunits with the canonical condensin, regulator of chromosome structure during mitosis and meiosis [107]. Condensin I^DC^ is composed of MIX-1, DPY-27 (DCC-specific), DPY-26, DPY-28, and CAPG-1 [6, 102, 103, 105–107]. The second part is a recruitment complex, composed of SDC-1, SDC-2, SDC-3, as well as two associated proteins DPY-21 and DPY-30 [6, 101, 104, 106, 108]. The high degree of similarity to condensin has led to the hypothesis that dosage compensation in the worm is achieved by a change in X chromosome structure. 

**Figure 4:**
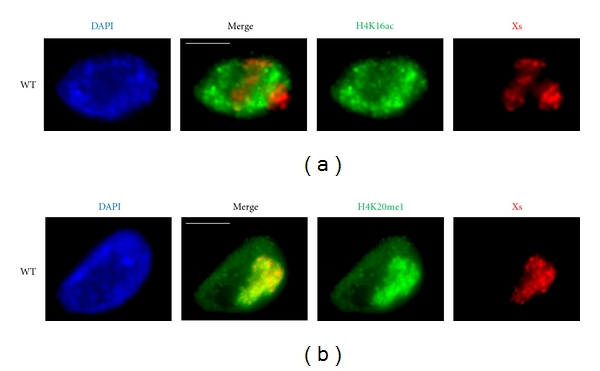
H4K16ac is reduced, and H4K20me1 is enriched, on the X chromosomes in WT hermaphrodite *C. elegans*. Shown are representative immunofluorescence projection images. (a) H4K16ac (green) is markedly reduced on the WT hermaphrodite X chromosomes (red, marked with anti-SDC-3 (DCC) antibodies). (b) H4K20me1 (green) is prominently enriched on the WT hermaphrodite X chromosomes (red, marked by anti-CAPG-1 (DCC) antibodies). DNA (DAPI) is shown in blue. Scale bars are 5 microns in length.

Recent work has identified several connections between chromatin modifications and the DCC. The histone H2A variant, HTZ-1 (H2A.Z), plays a role in DCC localization. Loss of *htz-1 *did not alter expression of DCC components, but instead led to spreading of the DCC to autosomes [109]. A survey of histone modifications using ChIP-chip analysis by the modENCODE project found an enrichment of H4K20me1 on the X chromosomes [110, 111]. Using immunofluorescence microscopy, we also observed enrichment of this mark on the X chromosomes in hermaphrodite somatic cells. Furthermore, we see a depletion of the mark antagonized by H4K20me1, H4K16ac. The hermaphrodite X chromosomes show sex- and DCC-dependent enrichment of H4K20me1 and underrepresentation of H4K16ac (Figure 4) (MW and GC, unpublished). Interestingly, worms seem to lack traditional K20 marks of constitutive heterochromatin, H4K20me2 and me3, but retain widespread H4K20me1 [112]. H4K20me2/3 are present in other major eukaryotes, including mammals and *Drosophila* [113]. Therefore, worm dosage compensation uses the same chromatin marks as the ones used in flies, but in opposite ways. In flies, upregulation of the X chromosome involves an enrichment of H4K16ac and may involve a depletion of H4K20me1. By contrast, in worms, downregulation of the X chromosomes may involve depletion of H4K16ac and enrichment of H4K20me1. It will be interesting to investigate in the future how these chromatin marks affect the transcription machinery in worms. 

### 6.3. Transcriptional Silencing: Mammals

Unlike flies and worms, which achieve dosage compensation by modulating transcription of the X chromosome(s) by an average of two-fold, the mammalian solution to dosage compensation is to silence one X chromosome in females. Many different chromatin marks play a role in X-chromosome inactivation (see below) [118]. X-chromosome inactivation occurs in therian mammals, which includes marsupials and placental mammals, but excludes monotremes. Female monotremes, or egg-laying mammals such as platypus, have stochastic inhibition of genes on the X [119] and no histone H4 modification differences between males and females or X chromosomes and autosomes [114]. Like chickens, monotremes may alter chromatin regionally, rather than chromosome-wide, to achieve gene-specific dosage compensation. Placental mammal and marsupial females have one pair of X chromosomes, and the male has an XY pair. In both placental mammals and marsupials, one X chromosome in the females is inactivated, resulting in both the female and male having one active X chromosome. 

X chromosome inactivation in marsupials is imprinted, and the paternal X is always the inactive X. The short arm (Xp) of the X chromosome is gene poor and heterochromatic. The long arm (Xq) is gene rich and is the dosage compensated part of the X chromosome [120]. The active X maintains high levels of H4 acetylation on the long arm, similar to the single male X, while the heterochromatic short arm has low levels of acetylation [115, 121]. Another study examined specific acetylation of H4K8 or H4K16 and discovered reduced acetylation of both chromatin marks on one female X chromosome in the majority of metaphases [114]. Other activating chromatin marks (H2AK5ac, H3K4me2, H3K9ac, and H4K8ac) are also reduced on the inactive X in marsupial females [114, 115, 122]. Therefore, in female marsupials, the inactive X chromosome is globally depleted of activating chromatin marks, and this depletion correlates with RNA Polymerase II exclusion from the X chromosome territory [122]. 

Unlike marsupials, female placental mammals randomly inactivate one X chromosome around the blastocyst stage of development. Aside from the choice of chromosome to inactivate (imprinted versus random), the overall mechanism of X-inactivation may seem similar between marsupials and placental mammals. However, there are some important differences. In placental mammals, a non-coding RNA *Xist* coats the inactive X chromosome and recruits chromatin modifying complexes that establish epigenetic marks. The *Xist* gene is present in all placental mammals analyzed, but is absent in marsupials, suggesting that chromosome-wide inactivation evolved first in a common ancestor, and *Xist* RNA, and the chromatin modifications it recruits added an extra layer of transcriptional repression [122–125]. 

The mammalian inactive X chromosome is marked by an array of chromatin modifications. Similar to the marsupial inactive X, the inactive X in placental mammals is generally depleted of activating chromatin marks. Histone H4 lysines 5, 8, 12, and 16 are hypoacetylated on the inactive X chromosome [116]. At the gene level, acetylation of specific H4 lysine residues can be detected at the promoters of X-linked genes on the active X chromosome; however there is little to no lysine acetylation of H4 at these genes on the inactive X chromosome [117]. The inactive X is also depleted of acetylation of H3 and H2A [126, 127] and H3 lysine 4 methylation [128]. Unlike the marsupial inactive, the inactive X in placental mammals is also characterized by an *Xist* RNA-dependent accumulation of repressive marks characteristic of facultative heterochromatin. H3K27me3 and the Polycomb complex member Ezh2 are also enriched on, and recruited to chromosomes expressing *Xist * [34, 129, 130]. Other repressive modifications, including monoubiquitination of H2AK119 and dimethylation of H3 lysine 9, also accumulate on the inactive X [131–134]. In a transgenic context, *Xist* RNA expression also triggers an increase in H4K20me1, independent of silencing, and therefore H4K20me1is proposed to be an early mark of X chromosome inactivation [34]. An increase in H4K20me1 was accompanied by a decrease in H4K16ac, consistent with an antagonistic relationship between these two marks [34]. However, a functional role for H4K20me1 or Pr-Set7 in X chromosome inactivation has not been demonstrated. These (or some of these) chromatin changes are thought to contribute to the formation of a repressive nuclear compartment devoid of RNA Polymerase II [135]. Therefore, the depletion of the H4K16ac and other activating chromatin marks in marsupials, as well as the depletion of these marks in combination with the accumulation of repressive marks (including H4K20me1) in placental mammals, leads to transcriptional silencing, an outcome very different from a two-fold modulation of transcriptional activity in flies and worms. 

## 7. Summary and Conclusions

Different mechanisms of dosage compensation have evolved to equilibrate expression of the X chromosomes between females and males and between the X and autosomes. The methods of dosage compensation that are most well understood include two-fold transcriptional upregulation in male flies, two-fold transcriptional downregulation in hermaphrodite worms, and transcriptional silencing in most mammals. 

The H4K16ac chromatin mark is either enriched or depleted on the dosage compensated X chromosomes in all three systems (Table 1). Where upregulation is required (in flies), H4K16ac is increased, which is proposed to contribute to chromosome decompaction, preventing chromatin remodeling by ISWI and allowing access of factors for productive elongation. A two-fold downregulation (in worms) may require the opposite: H4K16ac is reduced on the downregulated X chromosomes. Learning from the fly model, one may predict an increased role for ISWI in chromatin remodeling into a more repressive state and subsequently inhibited transcriptional elongation. Mammals sculpt the chromatin of the inactive X more drastically by creating more stable facultative chromatin that lacks activating marks, such as H4K16ac, and is enriched for repressive marks, such as H4K20me1. While the H4K16ac and H4K20me1 modifications are shared by all three mechanisms, mammals achieve more stable silencing when these marks are used in combination with other histone modifications.

How did these diverse dosage compensation mechanisms, with such different transcriptional outputs, evolve? Perhaps the reason for the difference is due to separate evolution of the dosage compensation machineries. The fly dosage compensation machinery coopted a conserved histone acetyltransferase complex [136]. In this organism, H4 acetylation of the X balances X-linked transcription between the sexes. Worms make use of a condensin-like complex for their dosage compensation machinery, suggesting that dosage compensation may involve partial condensation of the X chromosome [105, 107]. Consistent with this idea, reduced H4K16ac contributes to chromatin compaction and results in decreased transcription (as discussed above). Mammals use depletion of H4K16ac in combination with depletion of other activating chromatin marks to achieve transcriptional silencing. In addition, placental mammals acquired the *Xist* long non-coding RNA. Non-coding RNAs have an established role in transcriptional silencing in many processes, including imprinting and X inactivation [137]. *Xist* RNA then serves to recruit chromatin-modifying activities, leading to the accumulation of repressive chromatin marks. Therefore, the same modification, H4K16ac, depending on the chromatin context, leads to vastly different transcriptional outputs.

## Figures and Tables

**Table 1:** Summary of H4K16ac and H4K20me1 modifications on the dosage compensated X chromosomes.

	Levels of histone modification on the dosage compensated X chromosome(s)			
	H4K16ac	References	H4K20me1	References
*Drosophila*	Enriched on male X	[81–84]	Low levels on male X	[30]
*C. elegans*	Depleted from hermaphrodite Xs	Figure 4; MW, GC (unpublished)	Enriched on hermaphrodite Xs	Figure 4; MW, GC (unpublished); [110, 111]
Therian mammals	Decreased on the inactive X	[34, 114–117]	Enriched on the inactive X	[34]
